# Lipid Modulating Anti-oxidant Stress Activity of Gastrodin on Nonalcoholic Fatty Liver Disease Larval Zebrafish Model

**DOI:** 10.3390/ijms20081984

**Published:** 2019-04-23

**Authors:** Owais Ahmad, Bing Wang, Kejian Ma, Yang Deng, Maoru Li, Liping Yang, Yuqi Yang, Jingyun Zhao, Lijun Cheng, Qinyang Zhou, Jing Shang

**Affiliations:** 1 School of Life Sciences and Technology, China Pharmaceutical University, Nanjing 211198, China; Owaisk90@outlook.com; 2 School of Traditional Chinese Pharmacy, China Pharmaceutical University, Nanjing 211198, China; weibowang@yeah.net (B.W.); youngd@yeah.net (Y.D.); limaoru2016@163.com (M.L.); 3 The Institution of Yunnan Traditional Chinese Medicine and Materia Medical, Kunming 650223, China; ynylp81@163.com (L.Y.); yqyang57@163.com (Y.Y.); 13888964089@163.com (J.Z.); 4 Zhao Tong University, Zhaotong 657000, China; chenglijun224@163.com; 5 College of Life Sciences, Hubei University, Wuhan 430062, China; ZQYTeddy@163.com

**Keywords:** non-alcoholic fatty liver disease, non-alcoholic steatohepatitis, larval zebrafish, Gastrodin

## Abstract

Non-alcoholic fatty liver disease (NAFLD) and nonalcoholic steatohepatitis (NASH) is the most common chronic liver disease in the world. However, there are still no drugs for NAFLD/NASH in the market. Gastrodin (GAS) is a bioactive compound that is extracted from *Gastrodia elata,* which is used as an active compound on nervous system diseases. Recent reports showed that GAS and *Gastrodia elata* possess anti-oxidant activity and lipid regulating effects, which makes us curious to reveal the anti-NAFLD effect of GAS. A high cholesterol diet (HCD) was used to induce a NAFLD larval zebrafish model, and the lipid regulation and anti-oxidant effects were tested on the model. Furthermore, qRT-PCR studied the underlying mechanism of GAS. To conclude, this study revealed a lipid regulation and anti-oxidant insights of GAS on NAFLD larval zebrafish model and provided a potential therapeutic compound for NAFLD treatment.

## 1. Introduction

Non-alcoholic fatty liver disease (NAFLD) is the most common chronic liver disease with a 25% prevalent around the world [1]; it is characterized as a pathological spectrum from simple steatosis to nonalcoholic steatohepatitis (NASH) and further deteriorate fibrosis, cirrhosis, and hepatoma [2,3]. However, there is still no drugs for NAFLD/NASH in the market. Multiple pathogenesis factors lead to therapeutic drugs development of NAFLD becoming demanding and insufficient. “Two-hit” theory is the classical pathogenesis of NAFLD, which believed that the hepatic overlord-lipid accumulation and the oxidant stress are the two main factors of NAFLD [4,5]. Therefore, therapeutic strategies for NAFLD mostly focused on hepatic lipid regulating and anti-oxidant.

**Figure 1:**
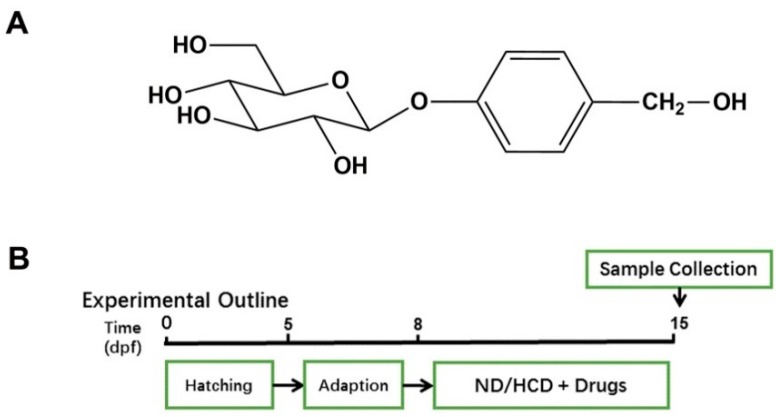
Effect of Gastrodin (GAS) on high cholesterol diet (HCD) induced larval zebrafish. (**A**) Chemical structure of GAS; (**B**) Experimental outline of the feeding protocol.

Gastrodin (GAS, Figure 1A) is a bioactive compound that is extracted from *Gastrodia elata* [6], which is an ancient clinical herb that is widely used for central disorders in China. GAS was widely reported as an active compound on nervous system diseases, such as headache, dizziness, spasm, epilepsy, stroke, amnesia, etc. [7]. Interestingly, recent reports showed that GAS and *Gastrodia elata* performed an anti-oxidant activity, lowering blood lipids and exhibiting hepatic lipid-lowering effects [8,9,10], which gives a hint that GAS could be a therapeutic compound for NAFLD.

Recently, larval zebrafish (*Danio rerio*) has become an attraction for *in vivo* model on drugs screening. With advantages of small size, physiological similarity to mammals, low cost, and being easy to maintain [11], larval zebrafish have been widely used in the study of lipid metabolism disorders related diseases [12,13], including NAFLD [14,15].

Based on the knowledge of GAS and zebrafish screening models, the present study focused on the lipid-regulating effect and anti-oxidant effect of GAS via using the high cholesterol diet (HCD) induced NAFLD larval zebrafish model. Furthermore, the underlying mechanism of GAS on anti-NAFLD was investigated.

## 2. Results

### 2.1. Effects GAS on HCD Induced Larval Zebrafish Model

**Figure 2:**
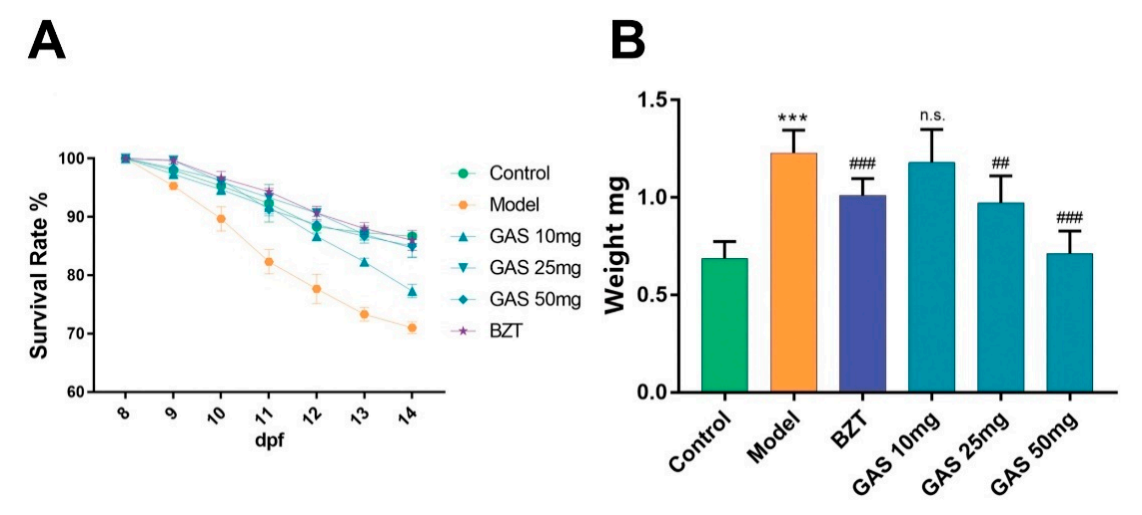
Effect of GAS on HCD induced larval zebrafish. (**A**) Mortality of larval zebrafish (n = 3); (**B**) Weight of larval zebrafish (n = 30). Bar indicate means ± SD. n.s. indicate no significant; *** *p* < 0.001 represent as compared with the control. ## *p* < 0.01, ### *p* < 0.001 represent compared with Model. *p* < 0.05 was considered to statistically significant, as calculated by One-way ANOVA, followed by Tukey’s test.

After ten days stimulation by HCD, the survival rate of larval zebrafish on model groups was found to continually decrease. However, after seven days’ administration of GAS and bezafibrate (BZT), the survival rate of the GAS and BZT groups decreased slower than model groups. Interestingly, the decrease ratio of GAS 25 mg, GAS 50 mg, and BZT group was nearly the same as control groups. The GAS attenuated the mortality of HCD induced larval zebrafish (Figure 2 A). Moreover, the weight of the larval zebrafish was tested, and Figure 2B shows the result. The weight of larval zebrafish in model groups significantly increased when compared to control, the weight of larval zebrafish in BZT groups decreased when compared with the model groups. Notably, the weight of larval zebrafish in GAS groups dose-dependently decreased as compared to the model group, indicating that the GAS took a weight-losing effect on HCD induced larval zebrafish (Figure 2B).

### 2.2. Lipid Regulating Effect GAS on the Larval Zebrafish Model

**Figure 3:**
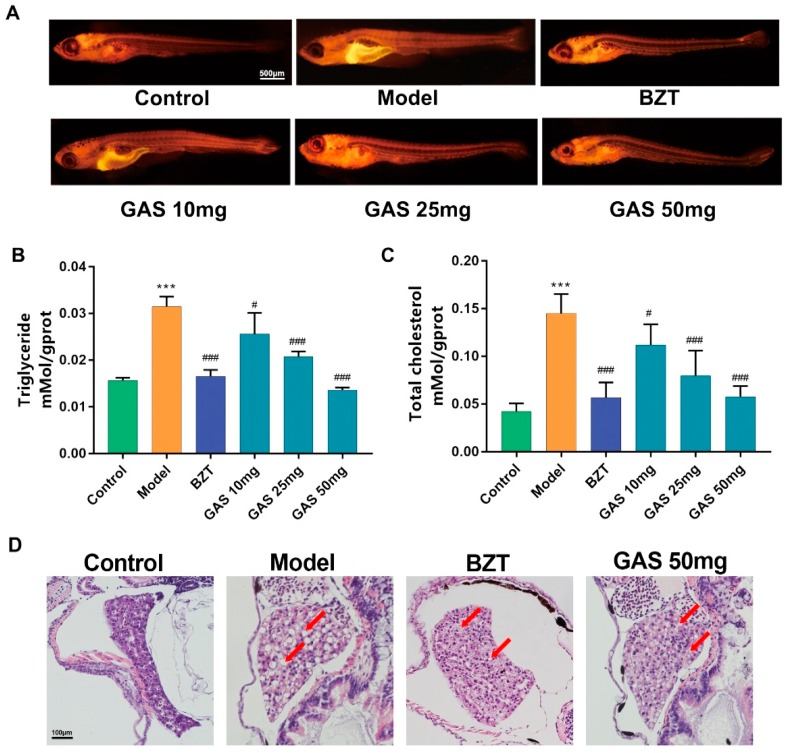
Lipid is regulating the effect of GAS on HCD induced larval zebrafish. (**A**) Nile red stain of larval zebrafish; (**B**) Triglyceride (TG) levels; and (**C**) total cholesterol (TC) levels of larval zebrafish in each group. (**D**) hematoxylin and eosin (HE) staining of larval zebrafish liver, macrovesicular steatosis and the differences mentioned with red arrows. Bar indicate means ± SD. *** *p* < 0.001 represent compared with the control. # *p* < 0.05, ### *p* < 0.001 represent compared with Model. *p* < 0.05 was considered as statistically significant, calculated by One-way ANOVA, followed by Tukey’s test. (n = 30).

The Nile red stain was performed on larval zebrafish to reveal the lipid-regulating the effect of GAS on HCD induced larval zebrafish. Nile red is a red phenoxazone dye lipid fluorescent dye, which can label the neutral lipid properties [16]. As Figure 3A shows the Nile red results, the fluorescence intensity and area increased significantly on the model groups as compared to the control. However, both the intensity and the area of red fluorescent decreased on the GAS and BZT groups. Notably, the intensity of red fluorescent on GAS groups was dose-dependently reduced. The triglyceride and total cholesterol of larval zebrafish were further tested. As the results are shown in Figure 3B,C, the HCD increased both Triglyceride (TG) and total cholesterol (TC) on the model groups. BZT, as a well-known lipid to regulate drugs, decreased both the TG and TC of HCD induced larval zebrafish. Notably, the GAS significantly dose-dependently decreased both TG and TC (Figure 3B,C). GAS is shown to have a lipid regulating effect on HCD induced larval zebrafish, as taken above. The hematoxylin and eosin (HE) stain provided a reliable evidence on the evaluation of anti-hepatic steatosis effect of drugs. The results in Figure 3D suggest that, after feeding larval zebrafish with HCD for one week, the macrovesicular steatosis occupied over 50% to 70% of the liver, which can be identified as NAFLD. The differences are marked with a red arrow. In the control, part of Figure 3D cleared from macrovesicular steatosis. In the model, it is easy to identify the macrovesicular steatosis, with the treatment with BZT and GAS almost showing similar results. 

### 2.3. Anti-oxidant Stress Effect GAS on the Larval Zebrafish Model

**Figure 4:**
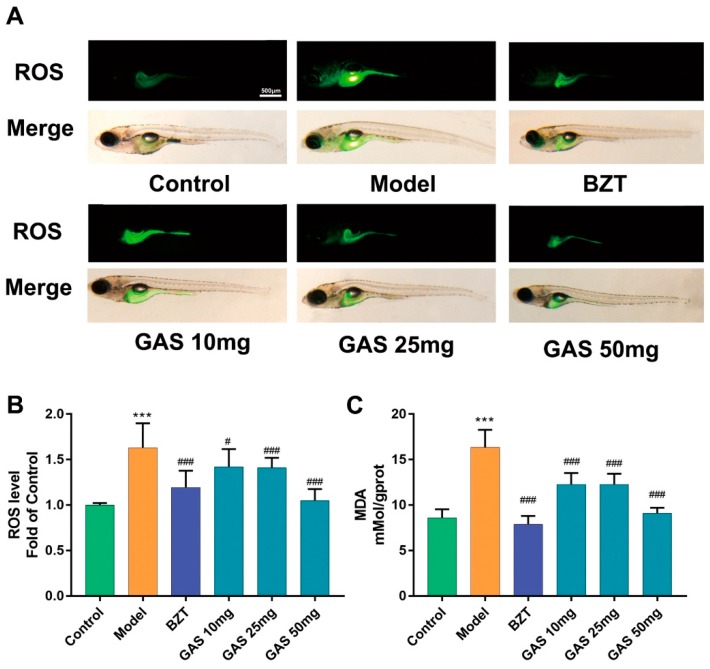
The anti-oxidant stress effect of GAS on HCD induced larval zebrafish. (**A**) The ROS production showed in fluorescence image and merged with a light field image. (**B**,**C**) Quantitation of reactive oxygen species C malondialdehyde (ROS. C. MDA) of each treated larval zebrafish group. Bar indicate means ± SD. *** *p* < 0.001 represent compared with the control. # *p* < 0.05, ### *p* < 0.001 represent compared with Model. *p* < 0.05 was considered as statistically significant, as calculated by One-way ANOVA followed by Tukey’s test. (n = 30).

To reveal the anti-oxidant effect of GAS on HCD induced oxidant attack on larval zebrafish, 2′,7′-dichlorofluorescein diacetate (DCFH-DA), which is a fluorescent dye of ROS, was used to stain the oxidant stress product ROS on larval zebrafish. From the staining results (Figure 4A), the fluorescence was increasingly distributed in the abdomen of larval zebrafish on model groups as compared to the control; however, in BZT and GAS groups, the fluorescence significantly decreased. Furthermore, a commercial assay, which was used to test the relative quantification of ROS level, showed a markedly higher in the HCD group than control (Figure 4B). The measurement of malondialdehyde (MDA) level showed a significantly higher level in the HCD group than the control group (Figure 4C). Notably, all of the GAS groups showed a dose-dependent improvement on ROS and MDA.

### 2.4. mRNA Expression Changes GAS on the Larval Zebrafish Model

**Figure 5:**
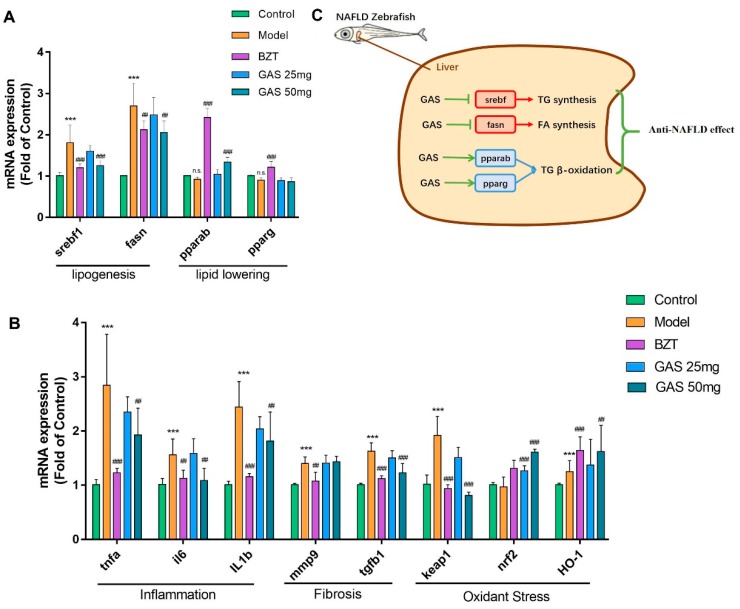
mRNA expression profile of GAS on HCD induced larval zebrafish and Molecular Mechanism of GAS. (**A**) mRNA expression of lipogenesis and lipid-lowering of larval zebrafish. (**B**) mRNA expression of inflammation, Fibrosis and oxidant stress of larval zebrafish. (**C**)molecular mechanisms of lipid metabolism modulation by GAS. Bar indicate means ± SD. n.s. indicate no significant; *** *p* < 0.001 represent compared with the control. # *p* < 0.05, ### *p* < 0.001 represent compared with Model. *p* < 0.05 was considered as statistically significant, as calculated by One-way ANOVA followed by Tukey’s test. (n = 6).

We performed a real-time qRT-PCR experiment to test the mRNA expression changes of GAS on lipogenesis, lipid-lowering, inflammation, fibrosis, and oxidant stress to further reveal the underlying mechanism of GAS on multiple pathogenesis aspects of NAFLD. As shown (Figure 5A), both lipogenesis related gene Sterol-regulatory element binding proteins (srebf1) and fatty acid synthase (fasn) were significantly increased on the model group and pulled back by GAS and BZT as compared with the control (Figure 5A). Lipid-lowering related gene peroxisome proliferator-activated receptor alpha (pparab) were increased in both drugs groups when compared with the model (Figure 5A). However, in GAS groups, the peroxisome proliferator-activated receptor gamma (pparg) did not show any changes when compared to the model (Figure 5A). Moreover, from inflammation mRNA expression result, all three-inflammation gene tumor necrosis factor alpha (tnfa), interleukin 6 (il6), and interleukin 1 beta (il1b) were increased extremely significantly in the model group and reduced by both drugs groups (Figure 5B) as compared with the control. Furthermore, one of the two fibrosis-related gene expression transforming growth factor-β (tgfb1) was significantly increased and pulled back by GAS, but the other matrix metalloproteinase 9 (mmp9) did not (Figure 5B). Finally, one of the gene kelch-like ECH-associated protein 1 (keap1) was increasingly expressed on the model group and pulled back by both drugs when compared with the control (Figure 5B). The other two anti-oxidant related genes nuclear factor-like 2 (nrf2) and heme oxygenase 1 (HO-1) were significantly increased in both drugs groups when compared with the control (Figure 5B). As shown (Figure 5C), both lipogenesis related gene Sterol-regulatory element binding proteins (srebf1) and fatty acid synthase (fasn) were pulled back by GAS. Lipid-lowering related gene peroxisome proliferator-activated receptor alpha (pparab) were increased in GAS. However, in GAS groups, the peroxisome proliferator-activated receptor gamma (pparg) did not show any changes. Taken together, GAS performed the lipid-regulating effect by improving srebp1, fasn, and pparab mRNA expression; furthermore, GAS also improved the inflammation, fibrosis, and oxidant stress gene expression.

## 3. Discussion

The present results demonstrate the lipid regulate effect and the anti-oxidant effect of GAS on HCD induced NAFLD larval zebrafish model. The phenotype on lipid metabolism, lipid-induced oxidant stress, and investigation of the underlying mechanism were carried out to reveal the anti-NAFLD effect of GAS.

The development of larval zebrafish from 5dpf to 15dpf always has natural mortality [17]. Moreover, a pathogenic factor, like HCD, will increase the death of larval zebrafish (Figure 2A). These changes make the mortality as a sensitive index to evaluate the effect of the drug. In the result, both the GAS and BZT improved the mortality. Obesity and overweight have turned out to be high-risk factors of NAFLD, which performed as a lipid accumulation on the whole body [18]. As Figure 2B shows the results, GAS reduced the weight of larval zebrafish that gained the weight by HCD. In summary, GAS improved the mortality and weight increment on HCD induced NAFLD larval zebrafish.

It is possible to locate the whole body lipid distribution by using lipid stain material on larval zebrafish due to the transparency of larval zebrafish. From the results (Figure 3A), after administration of HCD, the lipid increased in the whole body of the zebrafish, particularly in the abdomen of larval zebrafish, where the liver and adipose tissue are located. GAS successfully decreased the lipid accumulation on the abdomen of larval zebrafish, combined with the results of the TC and TG level (Figure 3B,C), it is shown that GAS could reduce the lipid accumulation on HCD induced NAFLD larval zebrafish. From the HE results (Figure 3D), it was identified that GAS has positive treatment effects as BZT.

Oxidant stress plays a crucial role in NAFLD [19]; the overlord oxidant stress production of ROS will virtually damage the liver cell [20]; furthermore, lipid peroxidation production MDA induces the progress of liver steatosis, resulting in the further deterioration of liver, which is believed as the “second hit” of NAFLD pathogenesis theory [21]. When combined with the results of lipid stain (Figure 3A) and the ROS stain (Figure 4A), the lipid accumulation area was as same as the area of increasing ROS. Therefore, the overlord lipid produced the overlord ROS, which could cause further deterioration of the liver. As Figure 3 shows the results, GAS reduced both ROS and MDA on the HCD induced larval zebrafish.

Whole genome sequencing of zebrafish was been finished in 2000 [22], which makes it possible to perform a gene expression test on larval zebrafish models for mechanism research. We further tested the mRNA changes of lipogenesis, lipid-lowering, inflammation, fibrosis, and oxidant stress on the larval zebrafish model to reveal the underlying mechanism of GAS on anti-NAFLD. The metabolism of lipid and fatty acids is relying on the balance of synthesis and β-oxidation [23]. Srebf1 and fasn are the two essential regulate gene of TG and fatty acid synthesis. For β-oxidation, the pparab and pparg effectively regulated the gene. From the results shown (Figure 5A,C), GAS improved both synthesis related gene srebf1 and fasn.

Moreover, GAS activated the pparab expression, which is the popular effect target of BZT on promoting lipid decomposition. However, there are no changes in pparg in the GAS groups (Figure 5A,C). A recent study showed that paprab is an actual therapeutic target for NAFLD [24], which give us a hint that GAS may take the anti-NAFLD effect by activating the PPARα pathway. The increased expression of inflammatory cytokines is believed to be the essential phenomena of the liver deterioration from NAFLD to NASH [25], and the anti-inflammatory strategy is widely used on the therapeutic of NAFLD [26]. From the results shown (Figure 5B), GAS reduced all three inflammatory cytokines expression, respectively.

Furthermore, hepatic fibrosis is believed to be the further deterioration of NASH, which is the principal factor of progression on NAFLD to hepatoma [27]. Notably, the GAS reduced the fibrosis gene tgfb1, which is the critical regulator of pro-fibrogenesis (Figure 5B). Keap1-Nrf2 is an oxidant regulate pathway that performs the activation of antioxidant enzyme systems.

Moreover, Nrf2 and HO-1 have been widely reported as the regulating genes of redox homeostasis [28,29] and as the potential target of the NAFLD in the liver. Notably, GAS reduced the keap1 expression, which is a sensor of ROS [30]; the result further verified the result of ROS (Figure 4A,B). Moreover, GAS increased both nrf2 and HO-1 mRNA expression, which indicate that GAS possibly takes the anti-oxidant effect by activating the anti-oxidant enzyme system [31]. In summary, GAS reduced the lipogenesis, inflammation, fibrosis, and oxidant stress mRNA expression, and it increased the lipid-lowering and anti-oxidant mRNA expression.

In conclusion, the present study demonstrates that GAS has both lipid-regulation and anti-oxidant effects on the HCD induced NAFLD larval zebrafish model. GAS reduced the mortality and weight of HCD induced larval zebrafish. Furthermore, GAS reduced the TG, TC, ROS, and MDA level on the larval zebrafish model. The possible underlying mechanism of anti-NAFLD effect of GAS is the suppression of srebp1, fans, tnfa, il6, il1b, tgfb, and keap1. The PPARαpathway and Nrf2, HO-1 pathway could be the possible effect target of GAS on lipid regulation and anti-oxidant. This study reveals the lipid regulation and anti-oxidant function of GAS and it provides a potential therapeutic compound for NAFLD treatment.

## 4. Materials and Method

### 4.1. Reagents

Cholesterol (92.5%), 2′,7′-dichlorofluorescein diacetate (DCFH-DA) was purchased from Sigma-Aldrich (St. Louis, USA). Bezafibrate (98%) and Nile Red (95%) was purchased from Aladdin (Shanghai, China). GAS (analytical standard, purity >99%) was obtained from National Institutes for Food and Drug Control of China (Beijing, China).

### 4.2. Preparation High Cholesterol Diet and Drug Solutions

The primary food for larval zebrafish (AP100) was purchased from Zeigler (PA, USA). The high-cholesterol diet (HCD) was prepared by mixing cholesterol with basic food. The final concentration of cholesterol in HCD was 5% (w/w). For drug solutions, DMSO was used to dissolve the drugs first, due to the low solubility of bezafibrate (BZT) in water, followed by dilution in DMSO solution with water to a final drug concentration of 10 μM/L (DMSO 0.001% v/v) for the administration of zebrafish. GAS was directly solved in water and each group was administrated with DMSO to the same concentration at 0.001% (v/v).

### 4.3. Maintenance Larval Zebrafish and Treatment

The zebrafish embryos were generated by natural spawning from parent wild-type AB-line adult zebrafish. After three days adaption from five-day post fertilization (dpf), larval zebrafish were randomly divided into six groups (n = 100 for each group), as follows: (1) Control group, fed with Normal diet (ND). (2) HCD group, fed with HCD (20 mg/tank per day). (3) BZT groups, fed with HCD (20 mg/tank per day) and BZT (5 mg/L), (4) GAS (10 mg/L), GAS (25 mg/L), and GAS (50 mg/L) groups, fed with HCD (20 mg/tank per day) and GAS (10 mg/L, 25 mg/L and 50 mg/L). All of the groups were maintained following the schedule that is shown in Figure 1B. The Science and Technology Department of Jiangsu Province approved all of the animal experiments and followed the Jiangsu Provincial standard ethical guidelines for the use of experimental animals under the ethical committees mentioned above.

### 4.4. Biochemical Measurement

Triglyceride (TG) levels, total cholesterol (TC) levels, and malondialdehyde (MDA) level were measured by commercial assay kits (Jiancheng, Nanjing, China), following the manufacturer’s instructions. The Reactive Oxygen Species Assay Kit (BeyoTime, China) detected the quantitation of reactive oxygen species (ROS), following the manufacturer’s instructions. All quantitation of the above kits was read by a multifunctional microplate reader (BioTek, USA).

### 4.5. Fluorescence Photography

Nile red is a lipophilic fluorescence material that can stain the TG and fatty acid. It can be detected at 543 nm (excitation wavelength) and 598 nm (scattering light). DCFH-DA is an indicator of ROS. DCFH-DA do not have any fluorescence property; only when ROS is oxidizing will it will perform fluorescence property by its oxidized product. Nile red was dissolved in acetone to prepare a 2 mg/mL solution. 1 mg/mL final concentration solution was diluted using water. The zebrafish larvae were stained in the dark for 30 min; then, we washed the zebrafish with water three times. After cleaning, the zebrafish were anesthetized with 0.05% tricaine and kept into the CMC-Na (4%). The image was immediately captured using a fluorescence stereoscope (Olympus SZX16). All of the captures were taken with the same parameters (exposure time, ISO and aperture) between different groups for comparison and all the above procedures were carried out in the dark. 

### 4.6. Histopathological examination

Larvae were fixed overnight with 4% paraformaldehyde (PFA) according to standard procedures and embedded in paraffin. 4 mm slide were stained with hematoxylin and eosin (HE) and captured on a light microscope (Olympus, Tokyo, Japan).

### 4.7. Real-time Quantitative PCR (qRT-PCR) Analysis

A total of 30 larval zebrafish of each group were sacrificed for the extraction of total RNA using Trizol reagent (Invitrogen, USA). HiScript II qRT SuperMix (Vazyme, China) performed reverse transcription for the synthesis of cDNA. The qPCR was performed on the StepOnePlus Real-Time PCR System (Applied Biosystems, USA) by adding the ChamQTM Universal SYBR qPCR Master Mix (Vazyme, China) and while following the manufacturer’s protocol. General Biotech Co., Ltd (Shang Hai, China) synthesized the specific sequences of primers used in this study and they are shown in (Table 1). The 2-∆∆Ct method was used to calculate the expression levels of each targeting mRNAs by normalized to GAPDH. 

### 4.8. Statistical Analysis

All of the data are expressed as mean ± SD. Graph Pad PRISM (Graph Pad Software, USA) was used for comparing the treatment group and the corresponding control by One-way ANOVA, followed by Tukey’s test for the significant difference. The differences between groups were considered to be statistically significant at *p*-value < 0.05.

## Figures and Tables

**Table 1:** Specific sequences of primers used in Real-time Quantitative PCR (qRT-PCR).

Gene Name	Acceccion Number (*Danio rerio*)	Forward Primer (5′->3′)	Reverse Primer (5′->3′)
*Danio Rerio*			
**srebf1**	NM_001105129	CATCCACATGGCTCTGAGTG	CTCATCCACAAAGAAGCGGT
**fasn**	XM_005169478	ATCTGTTCCTGTTCGATGGC	AGCATATCTCGGCTGACGTT
**pparab**	NM_001102567	CGTCGTCAGGTGTTTACGGT	AGGCACTTCTGGAATCGACA
**pparg**	NM_131467	CTGCCGCATACACAAGAAGA	TCACGTCACTGGAGAACTCG
**tnfa**	NM_212859	GCTTATGAGCCATGCAGTGA	TGCCCAGTCTGTCTCCTTCT
**il1b**	NM_212844	TGGCGAACGTCATCCAAG	GGAGCACTGGGCGACGCATA
**il6**	NM_001261449	AGACCGCTGCCTGTCTAAAA	TTTGATGTCGTTCACCAGGA
**mmp9**	NM213123.1	GAAGCGTTACGGCTACGT	TTCCATGTCTGGCGAATAG
**tgfb**	NM_182873.1	CATAAGAGCCACAGACAGAAG	GTAGAGCGAGCGTAAACAG
**keap1**	NM_182864.2	CCAACGGCATAGAGGTAGTTAT	CCTGTATGTGGTAGGAGGGTT
**nrf2**	NM_182889.1	TTGTCTTTGGTGAACGGAGGT	CTCGGAGGAGATGGAAGGAAG
**HO-1**	NM_001127516.1	GCT CAA CAT CCA GCT CTT TGA GG	GAC AAA GTT CAT GGC CCTGGG A

Specific sequences of primers used in this study are shown in the table.
